# Cutaneous lymphadenoma with locally aggressive features

**DOI:** 10.1016/j.jdcr.2025.12.042

**Published:** 2026-01-07

**Authors:** Matthew Solomon, Evan Mak, Christine M. Lee, Christopher Edens, Emily Wong, Wendi Wohltmann

**Affiliations:** a Department of Dermatology, San Antonio Uniformed Services Health Education Consortium, Joint Base San Antonio-Lackland, Texas; b Uniformed Services University, Bethesda, Maryland; c Departments of Dermatology and Pathology, San Antonio Uniformed Services Health Education Consortium, Joint Base San Antonio-Lackland, Texas

**Keywords:** adamantanoid, adnexal, aggressive, benign, cutaneous, excision, lymphadenoma, metastatic, Mohs, neoplasm, rare, trichoblastoma, tumor

## Introduction

Cutaneous lymphadenoma (CL) is a rare adnexal tumor with approximately 96^1^ reported cases worldwide with 1 case of malignant transformation and metastasis to regional lymph nodes. We present a case of a 33-year-old man with a CL on his left eyebrow that showed rare features of rapid regrowth and deep growth into subcutaneous tissue.

## Case description

**Fig 1:**
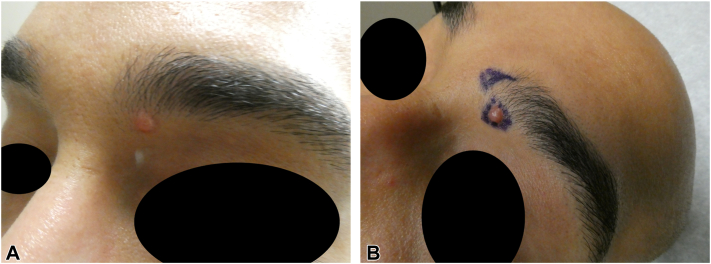
Photographs **(A, B)** of the lesion before biopsy showing round, dome shaped, pink firm papule with overlying atypical linear vessels.

**Fig 2:**
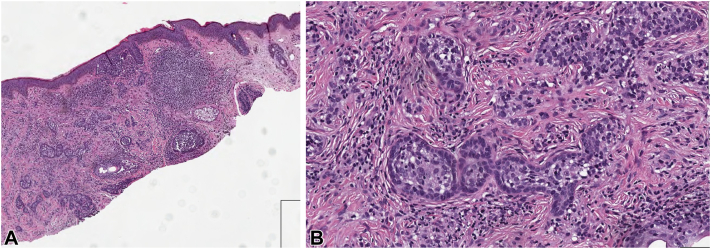
Shave biopsy demonstrates a proliferation of dermal nests and trabeculae composed of a peripheral, thin layer of basaloid cuboidal cells surrounding a central collection of epithelioid cells. The epithelioid cells have pale to clear cytoplasm, vesicular nuclei, and inconspicuous nucleoli, and lymphocytes are interspersed among these pale cells. The background dermis is fibrotic with scattered lymphohistiocytic inflammation. The dermal proliferation has a nodular appearance, though unencapsulated, and extends to the deep biopsy edge. (**A** and **B,** Hematoxylin-eosin stain; original magnifications: **A,** ×100; **B,** ×400.)

A 33-year-old man with no significant past medical history presented to the dermatology clinic with a small bump on his left eyebrow that began about 18 months prior. He initially thought it was a pimple that gradually grew but did not pop or drain as expected. He denied pain bleeding, drainage, itching, or any other symptoms. On physical and dermatoscopic examination, there was a 5 × 4 mm dome shaped, pink firm papule with overlying atypical linear vessels on the patient’s left medial eyebrow (Fig 1). No cervical lymphadenopathy was noted on examination. A shave biopsy of the lesion demonstrated a proliferation of dermal nests and trabeculae composed of a peripheral, thin layer of basaloid cuboidal cells surrounding a central collection of epithelioid cells (Fig 2).

A diagnosis of CL was made. The Mohs micrographic surgery appropriate use criteria requires additional clinical judgment for a rare biopsy-proven tumor. Mohs micrographic surgery was recommended to the patient based on recommendations and cases in the current literature.^2^

**Fig 3:**
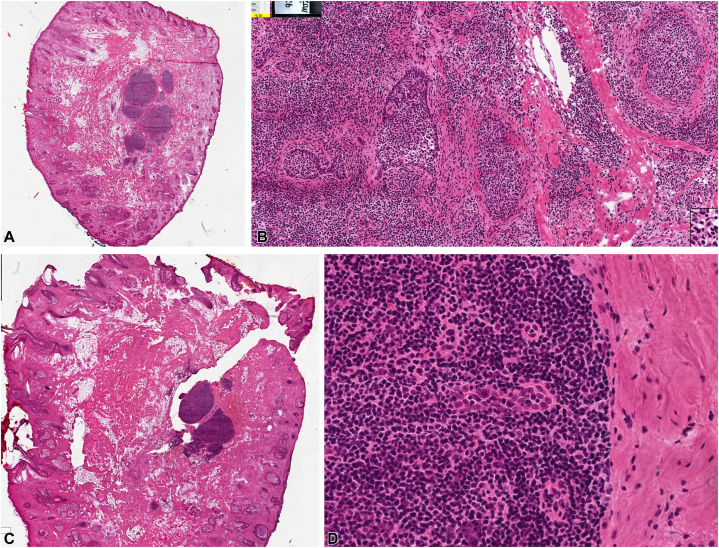
All slides in this figure are from stage 1 of Mohs micrographic surgery. **A, B,** Superficial sections. **C, D,** Deep sections. Histology of the superficial sections showed well-demarcated, large islands of cells in the dermis without significant atypia or atypical mitoses. Basaloid cells form a peripheral palisade without consistent tumor-stromal clefting. Some of the cells are larger with clear cytoplasm. There is a dense lymphocytic nodular infiltrate at the deep margin that could obscure tumor, however, there are tumor islands visible that are similar to those seen in the debulk. (**A-D,** Hematoxylin-eosin stain; original magnifications: **A,**×20; **B,** ×200; **C,** ×20; **D,** ×400.)

**Fig 4:**
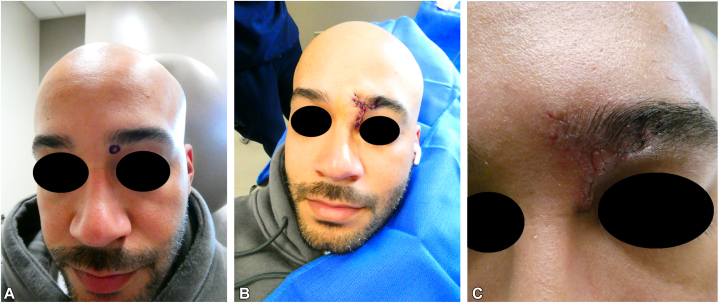
**A,** Photograph of the patient’s lesion before surgery. **B,** Photograph of patient immediately after Mohs micrographic surgery and advancement flap repair. **C,** Photograph after suture removal at follow-up appointment 7 days after surgery.

When the patient returned for surgery 4 months later, the lesion had grown to a size of 4 × 6 mm. The operation required 2 stages, as the first stage noted tumor deep in the section. The final depth of defect to achieve clear margins was muscle. Histopathology showed well-demarcated, large islands of cells in the dermis without significant atypia or atypical mitoses. Basaloid cells formed a peripheral palisade without consistent tumor-stromal clefting. Some of the cells were larger with clear cytoplasm, with scattered areas of lymphocytes. Dense lymphocytic nodular infiltrates were present as well (Fig 3). Repair with an advancement flap was successfully performed (Fig 4). The patient tolerated the procedure and postoperative 7 day follow-up period well. The patient reported no regrowth of the lesion at a follow-up telephone visit 22 months postoperative.

## Discussion

Our patient’s case of CL is notable because of the aggressive features of extending into deep cutaneous tissue and rapid regrowth after biopsy. CL is a rare skin neoplasm that presents on the head and neck of middle-aged adults.^3^ There are approximately 96 reports since its first documentation in 1987 when it was labeled lymphoepithelial tumor of the skin.^1^^,^^4^, ^5^, ^6^ Its initial clinical impression is often basal cell carcinoma because of similar clinical and histologic appearances. It is generally considered benign, with only 1 reported instance of malignant transformation of CL that metastasized to regional lymph nodes.^3^^,^^7^ This suggests that CL may have the potential to progress to a metastatic neoplasm if not completely excised.^1^ Because of this potential, it is important to be able to recognize and treat CL. Typical histology of CL is a well-circumscribed, unencapsulated nodule of basaloid lobules with a peripheral palisading array and lymphocyte-predominant infiltration.^1^^,^^7^ Notable features are islands of epithelial cells, with focal palisading embedded in fibrous loose-to-dense stroma as well as mature lymphocytes concentrated in the islands and scattered in the stroma.^1^^,^^3^^,^^8^ Our patient’s CL histology from the biopsy and excision was consistent with these findings (see Figs 2 and 3). The general consensus and common histology findings confer that CL is a benign tumor with AR and CK15 expression.^1^

On top of fitting the typical histologic criteria of CL, our patient’s case of CL had a set of rare features, as (1) it grew back rapidly after the initial biopsy and (2) its depth of invasion was notably deep in the subcutaneous layer. This differs from the typically reported presentation, where CL is a slow growing tumor that extends into the dermis.^1^^,^^5^ Similar to our case, Rajabi et al^7^ reported a case of initial gradual growth followed by rapid regrowth after shave excision; their case also had a depth of invasion extending to the subcutaneous fat. The authors suggested the recurrence was due to insufficient margins after the shave excision. Based on our observations and review of the literature, achieving a sufficient margin is a viable method to minimize chance of recurrence and regrowth. Additionally, because the sample size of documented CL cases is so low, it is possible that a higher percentage of total lesions behave with tendencies of regrowth and deeper depth of invasion than are reflected in the current literature. These aggressive features of regrowth and deep invasion combined with one documented case of metastasis may influence the view of the current paradigm that CL is a completely benign neoplasm.

CL’s origin is largely unknown, with speculation that it is an immature sweat gland tumor with ductal differentiation, a pilar or eccrine differentiation of basal cell carcinoma, or is derived from adnexal structures.^1^^,^^9^^,^^10^ There is preliminary evidence that CL commonly contains epidermal growth factor receptor mutations.^1^^,^^9^^,^^10^ As of 2023, the World Health Organization classified CL as an adamantanoid variant of trichoblastoma.^1^^,^^9^ In summary, current evidence suggests CL is an epithelioid tumor potentially of adnexal origin.

In conclusion, CL is a rare and benign tumor that is potentially of adnexal origin with a standard treatment of excision. Our case highlights CL with locally aggressive features of rapid regrowth after biopsy and extension into deep subcutaneous tissue. These features strengthen recommendations to biopsy a lesion with CL in the differential and treat with excision.^2^ Although currently believed to be benign, CL is such a rare tumor that unexpected aggressive behaviors as seen in our case may be more common than reported. Although many lesions may present with similar clinical and histologic features, CL is distinguished by characteristic histopathologic findings. Accordingly, biopsy is warranted when the diagnosis is unclear. Two areas of research that deserve further exploration are understanding the histogenesis and malignant potential of CL.

## Conflicts of interest

None disclosed.
